# Dr. Samuel Ensign

**Published:** 1917-12

**Authors:** 


**Dr. Samuel Ensign,** Geneva Medical College, died at his home in Arborville, Neb., Aug. 19, of cerebral haemorrhage, aged 98. He was for many years a druggist.

